# Does maternal body mass index during pregnancy influence risk of schizophrenia in the adult offspring?

**DOI:** 10.1111/j.1467-789X.2011.00971.x

**Published:** 2012-06

**Authors:** G M Khandaker, C R M Dibben, P B Jones

**Affiliations:** 1 Department of Psychiatry, University of CambridgeCambridge, UK; 2 Cambridgeshire and Peterborough NHS Foundation TrustCambridge, UK; 3 West Suffolk Hospital, Suffolk Mental Health Partnership NHS TrustBury St Edmunds, UK

**Keywords:** Body mass index, obesity, pregnancy, schizophrenia

## Abstract

Maternal obesity in pregnancy has been linked with several adverse outcomes in offspring including schizophrenia. The rising prevalence of obesity may contribute to an increase in the number of schizophrenia cases in the near future; therefore, it warrants further exploration. We reviewed current evidence regarding maternal body mass index (BMI) in pregnancy and risk of schizophrenia in adult offspring.

We searched PubMed and Embase databases and included studies that were based on large and representative population-based datasets. A qualitative review was undertaken due to heterogeneity between studies.

Four studies with 305 cases of schizophrenia and 24,442 controls were included. Maternal obesity (pre-pregnant BMI over 29 or 30 compared with mothers with low or average BMI) was associated with two- to threefold increased risk of schizophrenia in the adult offspring in two birth cohorts. High maternal BMI at both early and late pregnancy also increased risk of schizophrenia in the offspring. Discrepant findings from one study could be attributable to sample characteristics and other factors.

The area needs more research. Future studies should take into account obstetric complications, diabetes, maternal infections and immune responses that might potentially mediate this association.

## Introduction

Psychotic mental disorders are severe and usually chronic neuropsychiatric illnesses of adult life; among these, schizophrenia is perhaps the most severe. Schizophrenia is common with a lifetime prevalence of about 1% (1). It usually starts in young adulthood, peak incidence between ages 15 and 24 years (2). According to the World Health Organization (WHO), schizophrenia is the eighth leading cause of Disability Adjusted Life Years worldwide in the age group 15–44 years (3). Schizophrenia is characterized by altered perceptions (hallucinations and delusions), disturbances in cognition and affect, and often marked impairment in functioning (4). Currently, it is conceived as a developmental disorder of neuronal connectivity (5,6), linked to mechanisms involving dopaminergic (7), and glutamatergic neurotransmission (8,9). As with many long-term physical conditions, schizophrenia has both genetic and environmental components to its cause (2,10).

Accumulating evidence suggests that chronic adult diseases, physical as well as neuropsychiatric, may have their origins in early life (11). Alterations in fetal development have been reported to be associated with increased risk of adult cardiovascular and metabolic disorders (12–14). There is evidence of alterations in early neurodevelopment in schizophrenia. Birth cohort studies have consistently reported delays in infant motor development and childhood cognitive deficit in future cases of schizophrenia (15,16). Increased risk of schizophrenia among individuals with low birth weight suggests that adult schizophrenia may be linked with perturbations in fetal development (17). A large literature also links various maternal and obstetric factors with an increased risk of adult schizophrenia (18). These include gestational diabetes, pre-eclampsia, placental abruption, pre-term birth, emergency caesarean section, birth asphyxia, etc. (2,18). These findings provide empirical support for the currently widely accepted neurodevelopmental hypothesis of schizophrenia that postulates abnormalities in early neurodevelopment as a possible cause of the disorder (19,20).

Maternal nutritional status and body composition have important implications for fetal growth and neurodevelopment (21,22). Impaired development, particularly mental performance, among adult offspring of mothers who were exposed to famine during pregnancy (Dutch Hunger Winter 1944–1945) is a classic finding in epidemiology (22). Subsequently, lower maternal body weight before pregnancy was reported to be associated with an increased risk of schizophrenia in the 1958 British birth cohort (23). While these findings implicate fetal malnutrition in the causation of this neurodevelopmental disorder, recent studies have reported associations between higher maternal pre-pregnant body mass index (BMI) and various adverse outcomes of pregnancy. These include pre-eclampsia (24), fetal death (25,26) and congenital malformations (27). A recent review also suggests associations between high pre-pregnancy and pregnancy BMI and a range of adverse neurodevelopmental outcomes in the offspring (28). In Western societies where the general nutritional status of the population is currently good, it has been suggested that high rather than low maternal BMI may be more likely to be associated with adverse outcomes (29).

Over the last few decades, the prevalence of obesity has risen across the world at an alarming rate both in men and women (30,31). Between 1976 and 2004, obesity prevalence among women aged 12–44 years in the United States has doubled, and the number having a BMI over 40 has tripled (32). Similarly, there has been two- to threefold increase in the prevalence of maternal obesity in the United Kingdom between 1990 and 2004 (33). Recent data also suggest that the increase in prevalence of obesity in developing countries may be particularly great among women (34). If there is, indeed, an association between high maternal BMI during pregnancy and schizophrenia in adult offspring, a rise in the prevalence of maternal obesity may contribute to a significant excess number of schizophrenia cases in the future. Therefore, exploration of this association is necessary to inform future research directions as well as public health interventions. In this paper, we aim to summarize the existing evidence regarding maternal BMI before or during pregnancy and risk of schizophrenia in the adult offspring from a review of population-based epidemiological studies.

## Methods

### Search strategy

We searched Medline-PubMed and Embase databases from their respective inceptions to November 2011 for studies that were based on human samples and published in the English language. Search terms included both indexing terms (e.g. MeSH) and free texts: ([schizophrenia OR psychotic disorder OR psychosis OR psychoses) AND (body mass index OR BMI OR obesity OR body weight OR weight OR life style) AND (mothers OR pregnancy OR prepregnancy OR gestational OR maternal]). We also hand searched reference lists of included studies.

### Study selection

Strict criteria were applied in order to ensure that included studies had both good internal and external validity. Included studies used (i) general population sample with prospective or retrospective design; (ii) prospective measures of maternal BMI during or before pregnancy; (iii) a defined outcome (schizophrenia, schizophreniform or schizoaffective disorder) in the offspring by contemporary ICD or DSM classification system of mental illness and (iv) population-based registers or hospital records to identify cases.

### Data extraction

GMK carried out electronic search, examined all titles and abstracts, and obtained full texts of potentially relevant papers. GMK and CRMD read the papers and applied inclusion criteria working independently. Both came up with the same set of studies for inclusion in the review. Data were extracted by GMK from the studies that met the inclusion criteria. We used the STROBE checklists for quality assessment of observational studies (35) (available from URL: http://www.strobe-statement.org). Each study was critically appraised with a focus on sampling, measurements of exposure and outcome, follow-up, attrition, analytic strategy, measures to reduce bias and take into account confounding and other methodological concerns.

### Data synthesis and rationale for not using meta-analysis

Included studies varied in outcome definition and analytic strategies. For example, the outcome studied in the 1966 Finnish birth cohort was schizophrenia. This study calculated risk of schizophrenia in the offspring of mothers with pre-pregnant BMI over 29 compared with that of mothers with BMI below 19. On the other hand, the Child Health Development Study (CHDS) cohort used a broader outcome definition of schizophrenia and spectrum disorder (SSD). This study divided mothers into four categories according to pre-pregnant BMI and calculated risk of schizophrenia in the offspring of mothers in these categories compared to offspring of mothers with average BMI (defined as 20.0–26.9). There was also difference in timing of exposure measurement between studies. While both birth cohorts used maternal pre-pregnant BMI, two other studies included in the review used maternal BMI taken during pregnancy. Due to such heterogeneity between studies, combining results in meta-analyses was not deemed suitable. Therefore, we report a descriptive qualitative review.

## Results

**Figure 1:**
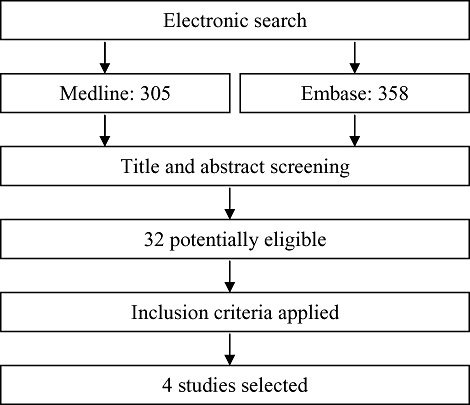
Selection of studies for the review.

We included four population-based studies of maternal BMI during or before pregnancy and schizophrenia in the offspring (Fig. 1 and Table 1). Together, these studies included data from 305 cases of schizophrenia and over 24,442 controls. Studies that did not meet the inclusion criteria, along with reasons as to why they were deemed ineligible, are presented in the Appendix.

**Table 1:** Population-based studies of maternal body mass index (BMI) and schizophrenia in adult offspring

Study	Design and setting	Case/control	Outcome and diagnostic criteria	Case identification	Age at follow-up (years)	Source of maternal BMI data
Jones *et al*. (1998) (36)	PC, Finnish 1966 birth cohort	76/10,502	Schizophrenia (DSM III R)	Hospital in- and out-patient attendance records	28	Records from midwife home visits and prenatal clinics
Schaefer *et al*. (2000) (39)	PC, subsample of CHDS cohort, United States	63/6,570	Schizophrenia and spectrum disorders (SSD) (DIGS)*	Hospital records, case-note review, and interview	30–38	Medical records and antenatal clinic records
Wahlbeck *et al*. (2001) (38)	RC, Helsinki, Finland	114/7,086	Schizophrenia, schizophreniform and schizoaffective (ICD or DSM)	Hospital discharge records	63–72	From birth records
Kawai *et al*. (2004) (42)	Case- control, Hamamatsu City, Japan	52/284	Schizophrenia (DSM IV)	Hospital in- and out-patient records	19	Mother and child handbook completed during pregnancy

* SSD includes schizophrenia, schizoaffective disorder, other non-affective psychosis and schizotypal personality disorder.

CHDS, child health and development study; DIGS, Diagnostic Interview for Genetic Studies; PC, prospective cohort study; RC, retrospective cohort study.

### Description of datasets

One study is based on the 1966 Finnish birth cohort (36), which is an unselected, general population sample ascertained during mid-pregnancy (37). It is composed of 12,068 pregnant women living in the provinces of Oulu and Lapland who were due to deliver during 1966. Their 12,231 children represent 96.3% of the births subsequently registered at the Finnish Central Statistical Office. The mothers of the remaining 3.7% either refused to participate or did not obtain prenatal care. Of the total 12,231 births, 12,058 were live births. The study included in this review is based on a representative risk set of 11,017 cohort members alive in Finland at age 16 (91% of all live births).

The risk set of another study is based on 27,068 births at the Helsinki University Central Hospital between 1924 and 1933 (38). The study only included children who also went to primary schools in the city of Helsinki. Both birth and school records were available for 8,580 individuals, out of which 7,086 still lived in Finland in 1971 who were included in the analysis (26% of all births).

Another report is based on a subsample of the Child Health and Development Study (CHDS) (39), which is a prospective birth cohort study of factors affecting outcomes of pregnancy and child development (40). Between 1959 and 1967, 19,044 live births to women who sought prenatal care in Alameda County, California, from the Kaiser Foundation Health Plan formed the CHDS cohort. In total, 12,576 children born to CHDS mothers were in contact with the Kaiser Foundation Health Plan until or after age 10 years and were potentially eligible for the study (41). Data on maternal pre-pregnancy BMI were available for 6,633 of these individuals who comprised the sample for the study included in this review (35% of all live births in CHDS).

The final study used a population-based case – control design, which included patients of schizophrenia born in one Japanese city (Hamamatsu) during or after 1966. Patients were collected from all four hospitals covering Hamamatsu and neighbouring areas. Controls were healthy volunteers living in the same residential areas as the cases and were also born after 1965. In addition to maternal BMI during early and late pregnancy, this study investigated frequency of antenatal clinic visits and obstetric complications in relation to schizophrenia in the adult offspring.

### Risk of schizophrenia

Two birth cohorts used maternal pre-pregnant BMI and reported associations in the same direction (Table 2). In the 1966 Finnish cohort, cumulative incidence of schizophrenia by age 28 years was 0.69% (95% CI 0.54%–0.86%) (36). Mean pre-pregnancy BMI of mothers of future cases and unaffected controls did not differ significantly. Further analysis compared the leanest (BMI < 19, *n* = 497) and the largest (BMI > 29, *n* = 524) fifth percentiles of mothers with the middle 90% (BMI 19.1–29, *n* = 8,640; reference group) in terms of risk of schizophrenia among adult offspring. In the group of mothers with BMI < 19, four children (0.8%) developed schizophrenia, similar to the rate (0.7%) in the reference group. However, among the offspring of mothers with BMI over 29, seven (1.3%) developed schizophrenia. Although the risk of schizophrenia was double among children born to mothers with pre-pregnant BMI > 29, this fell short of statistical significant after adjusting for gender of offspring, social class and age of mother at conception, odds ratio (OR) 2.1, 95% confidence interval (CI) 0.9–4.6; *P* = 0.07.

**Table 2:** Risk of schizophrenia in adult offspring of mothers with high body mass index (BMI) before or during pregnancy

Study and setting	Timing of BMI measurement	Risk of schizophrenia, Odds ratio (95% CI)	Other findings	Adjustment for confounding
Jones *et al*. (1998) (36), Finnish 1966 birth cohort	Pre-pregnancy	2.1 (0.9–4.6) for children of mothers with BMI > 29 compared with children of mothers with BMI 19.1–29	Significant association with schizophrenia and low birth weight (LBW), i.e. <2,500 g, LBW and short gestation (<37 weeks) combined, and perinatal brain damage	Gender of offspring, social class and maternal age at conception
Schaefer *et al*. (2000) (39), CHDS cohort, USA	Pre-pregnancy	2.9 (1.3–6.6) for children of mothers with BMI > 30 compared with BMI 20.0–26.9	–	Maternal age, ethnicity, parity, smoking, education
Wahlbeck *et al*. (2001) (38), Helsinki, Finland	Late pregnancy	3.75 (1.42–9.89) for children of mothers with BMI < 24 compared with BMI > 30	Significant association between LBW, length and placental weight and risk of schizophrenia.	–
Kawai *et al*. (2004) (42), Hamamatsu City, Japan	Early and late pregnancy	1.24 (1.02–1.50) for higher maternal BMI at early pregnancy and 1.19 (1.01–1.41) for late pregnancy	Number of antenatal care visit significantly associated with schizophrenia. Maternal BMI and number of antennal care visit associated with increased obstetric complications in case group	Birth order, gestational age of offspring

In the CHDS cohort, mean pre-pregnancy BMI was similar between mothers of the affected and unaffected subjects, but pre-pregnant weight was significantly higher in mothers of offspring with SSD (39). Risk of SSD was analysed using both categorical and continuous measures of BMI. First, mothers were divided into four groups according to their pre-pregnant BMI: below average (<19.9, *n* = 1,563), average (20.0–26.9, *n* = 4,441), above average (27.0–29.9, *n* = 364) and high (>30, *n* = 265). Compared with the group of mothers with average BMI (reference category), risk of SSD in the offspring of mothers with low BMI was not significantly different. Compared with the reference group, risk of SSD was higher (although not statistically significant) in the offspring of mothers with above average BMI, hazard ratio (HR) 2.0 (95% CI 0.85–4.8). However, a significant threefold increased risk of SSD was observed among children born to mothers with high pre-pregnant BMI (>30). Similar results were observed for narrowly defined schizophrenia (*n* = 38) (39). Further analysis using BMI as a continuous variable found pre-pregnant BMI was significantly associated with increased risk of both SSD (HR = 1.1; 95% CI 1.03–1.14) and narrowly defined schizophrenia (HR = 1.1; 95% CI 1.02–1.17). Maternal pre-pregnant BMI was not associated with age, the time of first hospitalization or outpatient treatment for schizophrenia in the adult offspring (39).

Two studies (from Helsinki and Hamamatsu) used maternal BMI that was measured during pregnancy and reported contrasting results. In the Helsinki study, cumulative incidence of schizophrenia was 1.6% (38). Contrary to the other studies, this study reported an increased risk of schizophrenia in the adult offspring for lower maternal BMI during late pregnancy. Using BMI as a continuous variable, risk of schizophrenia increased by 1.09 times (OR 1.09, 95% CI 1.02–1.17) for one unit decrease in maternal BMI. Using BMI as a categorical variable, over threefold increase in risk of schizophrenia was observed among children of mothers with BMI < 24 compared with children of mothers with BMI > 30.

In the Japanese sample, maternal BMI was measured at both early and late pregnancy (42). BMI was treated as a continuous variable. A 24% increase in risk of schizophrenia in the adult offspring was observed for one unit increase of maternal BMI during early pregnancy, which was statistically significant. Similar association (19% increased risk for one unit increase in BMI) was observed for maternal BMI in late pregnancy.

### Maternal characteristics

In both Finnish studies, age at the time of pregnancy and parity were similar between case mothers and mothers of unaffected controls (36,38). However, in the 1966 Finnish birth cohort, mothers of adult offspring with schizophrenia were more likely to smoke, report depressed mood and have diabetes during pregnancy than the control group (36). In the CHDS cohort, pre-pregnancy BMI was higher in women who were older at the time of delivery, came from an African – American ethnic background, had higher parity, fewer years of education and did not smoke (39). However, associations between pre-pregnant BMI and schizophrenia and spectrum disorders were essentially unaltered after adjustment for these factors (39). In the Japanese sample, case and control mothers did not differ significantly on age, physical illness or gestational age at the time of first and last antennal visit.

### Maternal BMI, fetal development and obstetric complications

From the selected studies, insufficient data were available on potential links between maternal BMI and fetal development and obstetric complications. Only one study specifically explored links between maternal BMI and obstetric complications. In the Japanese sample, the rate of obstetric complications was higher in case mothers compared with control mothers (OR 3.13, 95% CI 1.26–7.77). This is in line with previous studies (18). Frequency of maternal antenatal clinic visit was inversely associated with risk of schizophrenia in the offspring. However, there was evidence that increased maternal BMI during early pregnancy together with poor attendance of antenatal clinics, in part, contributed to the increased rate of obstetric complications in case mothers.

With regard to fetal development, in the Helsinki study, low maternal BMI in late pregnancy was associated with reduced placental weight and size at birth (38). The study also reported increased risk of schizophrenia for low birth weight (OR, 1.48 per kg; 95% CI, 1.03–2.13), shortness at birth (OR, 1.12 per cm; 95% CI, 1.03–1.22), and low placental weight (OR, 1.22 per 100 g; 95% CI, 1.04–1.43).

In the 1966 Finnish cohort, by contrast, mean birth weight and placental weight were similar between those who developed schizophrenia as adults and unaffected controls (36). However, low birth weight (<2,500 g) and combination of low birth weight and short gestation (<37 weeks) were more common among future cases of schizophrenia. Perinatal brain damage also increased risk of schizophrenia later in life. However, none of these variables were examined in relation to maternal BMI in this study.

## Discussion

Two birth cohort studies (1966 Finnish and CHDS, United States) reported over twofold increased risk of schizophrenia and spectrum disorder in adult offspring of mothers with high pre-pregnant BMI (over 29 or 30) compared with low or average BMI. One of these studies also reported significant increased risk of schizophrenia using maternal BMI as a continuous variable. However, one study reported an association in the opposite direction (i.e. lower maternal BMI and increased risk of schizophrenia in offspring). Unlike the birth cohorts that measured maternal BMI before pregnancy, this study used BMI in late pregnancy. As maternal BMI at different stages of pregnancy has been reported to be highly correlated (42), timing of BMI measurement is unlikely to be the explanation for the discrepant findings. Besides, the case – control study from Japan that measured maternal BMI in different stages of gestation reported that higher maternal BMI at both early and late pregnancy were associated with increased risk of schizophrenia in the adult offspring. However, other characteristics of the Helsinki sample may explain their discrepant findings.

The Helsinki study was based on a cohort of people born between 1924 and 1933 (38). In the 1930s, in Helsinki, about 60% of city's men were manual labourers. However, there was an excess of people from lower social class in this sample (78% manual labourers). Lower social class has been reported to be associated with schizophrenia, although whether it is a cause or consequence of illness is yet to be settled (2). Thus, in this study, it is possible that the observed association between lower maternal BMI and increased risk of schizophrenia in the adult offspring is confounded by lower social class. Other factors associated with poverty such as increased risk of infection may be also relevant. For example, adult schizophrenia has been reported to be associated with both childhood (43) and prenatal infection (44). These factors were not explored in this sample.

In addition, only being able to include cases of schizophrenia that were admitted to hospital after age 38 years may have introduced bias by over representation of chronic or late onset cases (38). Age at the time of follow-up was significantly older in the Helsinki study compared with the rest of the studies (Table 1). On the other hand, one study that included relatively early onset cases (average age 19 years) reported increased risk of schizophrenia for high maternal BMI both at early and late pregnancy (42). Whether early and late onset and/or chronic schizophrenia differ in terms of risk factor profile is a matter of debate. However, evidence suggests that early and late onset schizophrenia may be phenotypically different (45), and therefore, differences in risk factors are plausible.

In the Helsinki study, observed associations between schizophrenia and low maternal BMI in late pregnancy, low size at birth and low placental weight, all suggest to the authors that fetal malnutrition is a possible cause of the illness (38). While this is possible, according to the WHO ten-country study, incidence and prevalence of schizophrenia have not been reported to be excessively high in the developing countries where poverty and malnutrition is endemic (46). However, in support of the authors, one could argue that in the developing countries, many malnourished newborns may not survive until adulthood. Besides, the economy and lifestyle in Finland during the 1920s may have been vastly different from today. The Finnish civil war and the First World War left the economy fragile, leading to a large number of Finns immigrating to North America. As the authors also pointed out, a low maternal BMI in 1920s and 1930s in Finland is very likely to reflect severe malnutrition, poverty and illness (38). The rest of studies were all based on populations born after 1959 in affluent countries (including Finland), and all reported increased risk of schizophrenia for maternal obesity during or before pregnancy. This may be due to a shift in the overall nutritional status of the general population (47). It has been proposed in the current Western societies with increased availability of food that high rather than low maternal BMI may be more likely to be associated with adverse outcomes (29). Indeed, recent studies have linked higher maternal BMI during pregnancy with late fetal death (25,26), congenital malformations and other adverse outcomes of pregnancy (27).

Quality assessment of individual studies by STROBE checklists revealed that all studies gave adequate description of study participants, and measurement of exposure and outcome. However, only two studies adjusted their analyses for family history of psychotic illness (39,42). Data on pre-pregnancy weight were missing on 15% of mothers in one birth cohort (39). The authors did not include any comparison between mothers with missing data and those who were included in the study. The only case – control study included in this review was based on early onset cases of schizophrenia, the mean age of first psychotic symptoms is 14.1 years (SD = 2.6) (42). Therefore, results from this study may not be generalizable to all cases of schizophrenia.

The limited number of studies and the inability to include a meta-analysis due to the heterogeneity between studies make it difficult to draw a firm conclusion from this review. However, the included studies were based on large and representative population-based datasets and used robust methodology including prospective measurement of maternal BMI before or during pregnancy and follow-up in the offspring for schizophrenia. There is evidence of increased risk of schizophrenia in the adult offspring of mothers who were obese in pregnancy. Several factors could explain this finding.

High pre-pregnant BMI can increase risk of obstetric complications such as pre-eclampsia (24) and emergency caesarean section (48,49). Birth complications including emergency caesarean section, placental abruption, perinatal asphyxia, etc., are established risk factors for adult schizophrenia (18). Therefore, it is possible that increased risk of schizophrenia in the adult offspring of obese mothers is due to high rates of obstetric complications in these mothers. Indeed, in the Japanese sample, there was evidence that higher rate of obstetric complications in case mothers were partly attributed to high BMI (42). However, the rest of the studies did not account for obstetric complications in analyses of maternal BMI and risk of schizophrenia in the offspring.

Another possible mechanism underlying this association could be maternal gestational diabetes. Obesity is associated with increased risk of gestational diabetes (50). Poorly controlled maternal diabetes is also associated with an increased risk of neurodevelopmental abnormalities. These include neural tube defect (51) and impaired intellectual and psychomotor development in children (52). It has been suggested that deficiencies in certain micronutrients (53,54) and a sustained state of hyperglycemia and/or hyperinsulinemia (55), which are common in diabetes, may be responsible for such neurodevelopmental alterations. Maternal gestational diabetes has also been reported to be associated with increased risk of adult schizophrenia (18) and childhood non-clinical psychotic symptoms from birth cohort studies (56). In the 1966 Finnish birth cohort, case mothers were over seven times more likely than control mothers to have diabetes during pregnancy (although not statistically significant) (36). However, none of the studies adjusted for this potentially important mediator in their analysis.

More interestingly, in one cohort (CHDS), some overweight mothers were prescribed amphetamine just before or during pregnancy to avoid gaining ‘too much’ weight (39). Thus, association between high pre-pregnant BMI and risk of schizophrenia observed in this cohort may also be due to fetal exposure to this neurodevelopmental toxin (amphetamine) in some individuals (57).

Other mediating factors may also include maternal infection. A rich body of literature from ecological, prospective birth cohort and preclinical studies have linked maternal infection during pregnancy with increased risk of schizophrenia in adult offspring (44,58). Obese individuals are more susceptible to infections (59). In line with this suggestion, in the 1966 Finnish birth cohort, maternal fever >38°C (indicative of infection during pregnancy) was associated with a threefold increase in risk of schizophrenia in adult offspring (36).

As well as increasing susceptibility to infections, maternal obesity may also contribute to the risk of neurodevelopmental disorders through activation of the innate immune system and/or increasing levels of inflammatory cytokines. There is evidence that elevated levels of proinflammatory cytokines, such as interleukin-8 (IL-8) or tumour necrosis factor-alpha, in maternal blood during pregnancy can increase risk of schizophrenia in the adult offspring (human epidemiological studies) (60,61). Animal studies suggest that maternal simulated viral or bacterial infection or direct injection with IL-6 during pregnancy can give rise to intermediate phenotypes associated with schizophrenia in adult offspring (62). Some of these phenotypes such as deficits in sensory gating and latent inhibition (induced by prenatal exposure to IL-6) have been shown to be reversible by treatment with clozapine, an antipsychotic (63). There is evidence that proinflammatory cytokines, such as IL-6, can cross placenta (64) and blood – brain barrier (65). It has been suggested that IL-6 can affect neurodevelopment directly (66). They can also reprogramme the hypothalamic – pituitary – adrenal axis by increasing fetal exposure to maternal glucocorticoids (by inhibiting placental enzyme 11 beta-hydroxysteroid dehydrogenase type 2) (62,67–69). Dopaminergic over activity in the mesolimbic pathway and under activity in the mesocortical pathway have been reported in patients with schizophrenia (7). Prenatal immune activation in animals has also been linked with structural and functional alterations in the mesocorticolimbic dopaminergic system (70).

Obesity is associated with a chronic activation of the innate immune system and up-regulation of inflammatory cytokines, such as IL-6 (71). Moreover, white adipose tissue is a chief source of such inflammatory cytokines (71). BMI is likely to reflect body fat content. This may explain why a significant risk of schizophrenia was observed mostly among adult offspring born to obese mothers (BMI over 29 or 30) (36,39). It is possible, only at this level of obesity (or above), for sufficient inflammatory cytokine, such as IL-6 is available in maternal blood, to have any meaningful effect on fetal neurodevelopment. Whether the association between high maternal BMI during pregnancy and risk of schizophrenia in the adult offspring is indeed mediated by increased proinflammatory cytokines needs to be tested in future studies.

## Conclusion

Our review indicates that maternal obesity during pregnancy may be associated with increased risk of schizophrenia in the adult offspring. However, this review also points out other factors that may potentially mediate this association. These include obstetric complications, maternal infections and gestational diabetes, which needs to be addressed in future studies. We also offer a hypothesis on potential mechanisms involving the immune system for future investigations. Although associations between maternal obesity and various short-term adverse outcomes of pregnancy are well known, our review highlights a potentially long-term and severe consequence. Schizophrenia typically does not manifest until the third decade of life. Therefore, longer term results of the current obesity epidemic with regard to the incidence of schizophrenia may not be fully clear for some time to come. The scale of this potential problem should provide impetus for further work on this topic through collaborations between various clinical specialities, basic scientists and public health specialists.

## Conflict of Interest statement

GMK and CRMD report no competing interest. PBJ is co-inventor on patent PCT/GB2005/003279 (methods for assessing psychotic disorders). PBJ has received research support from GlaxoSmithKline and directs the National Institute for Health Research Collaborations for Leadership in Applied Health Research and Care for Cambridgeshire and Peterborough.

## Appendix

**Table:** Studies that did not meet inclusion criteria

No	Study	Reason for exclusion
1	Moilanen *et al*. Schizophrenia Research. 124(1–3), 2010	Another study from the same cohort on maternal pre-pregnancy BMI and schizophrenia in the adult offspring included in this review
2	Abel *et al*. Arch Gen Psychiatry. 67(9), 2010	Did not include any measure of maternal weight or BMI during or before pregnancy
3	Indredavik *et al*. Journal of Developmental and Behavioral Pediatrics. 31(4), 2010	Did not include any measure of maternal weight or BMI during or before pregnancy
4	Lahti *et al*. British Journal of Psychiatry. 195(2), 2009	Did not include any measure of maternal weight or BMI during or before pregnancy
5	Thomas *et al*. British Journal of Psychiatry. 194(6), 2009.	Outcome investigated was non-clinical psychotic symptoms at age 12 years, not adult schizophrenia
6	Insel *et al*. Arch Gen Psychiatry. 65(10), 2008	Did not examine maternal pre-pregnancy BMI in relation to schizophrenia in the adult offspring; another report from this cohort examined this association and was included in this review
7	Malaspina *et al*. BMC Psychiatry. 8, 2008	Did not include any measure of maternal weight or BMI during or before pregnancy
8	Khashan *et al*. Arch Gen Psychiatry. 65(2), 2008	Did not include any measure of maternal weight or BMI during or before pregnancy
9	Haukka *et al*. Psychological Medicine. 38(1) (pp 63–70), 2008	Did not include any measure of maternal weight or BMI during or before pregnancy
10	Ellman *et al*. Schizophr Res. 93(1–3), 2007	Did not include any measure of maternal weight or BMI during or before pregnancy
11	Watson *et al*. Eur J Clin Nutr. 61(11), 2007	Did not include any measure of maternal weight or BMI during or before pregnancy
12	Laursen *et al*. J Clin Psychiatry. 68(11), 2007	Did not include any measure of maternal weight or BMI during or before pregnancy
13	Bresnahan *et al*. Int J Epidemiol. 36(4), 2007	Did not examine maternal pre-pregnancy BMI in relation to schizophrenia in the adult offspring; another report from this cohort examined this association and was included in this review
14	McGrath *et al*. Schizophr Res. 81(1), 2006	Did not include any measure of maternal weight or BMI during or before pregnancy
15	Gunnell *et al*. Schizophrenia Research. 79(2–3), 2005	Did not include any measure of maternal weight or BMI during or before pregnancy
16	Spauwen *et al*. Acta Psychiatr Scand. 110(5), 2004	Did not include any measure of maternal weight or BMI during or before pregnancy
17	Gunnell *et al*. American Journal of Epidemiology. 158(4) (pp 291–300), 2003.	Did not include any measure of maternal weight or BMI during or before pregnancy
18	Bersani *et al*. J Matern Fetal Neonatal Med. 14(1), 2003	Did not include any measure of maternal weight or BMI during or before pregnancy
19	Cannon *et al*. Arch Gen Psychiatry. 59(1), 2002	Did not include any measure of maternal weight or BMI during or before pregnancy
20	Gunnell *et al*. Br J Psychiatry.181, 2002	Did not include any measure of maternal weight or BMI during or before pregnancy
21	Matsumoto *et al.* Psychol Med. 31(5), 2001	Did not include any measure of maternal weight or BMI during or before pregnancy
22	Ichiki *et al.* Psychological Medicine. 30(3), 2000	Used mean maternal body weight during pregnancy, not body mass index
23	Rosso *et al.* Schizophr Bull. 26(2), 2000	Did not include any measure of maternal weight or BMI during or before pregnancy
24	Seidman *et al.* Schizophrenia Bulletin. 26(2), 2000	Did not include any measure of maternal weight or BMI during or before pregnancy
25	Hultman *et al.* BMJ. 13; 318(7181), 1999	Did not include any measure of maternal weight or BMI during or before pregnancy
26	Hultman *et al.* Br J Psychiatry. 170, 1997	Did not include any measure of maternal weight or BMI during or before pregnancy
27	Wright *et al.* American Journal of Psychiatry. 152(12), 1995	Did not include any measure of maternal weight or BMI during or before pregnancy
28	Sacker *et al.* Br J Psychiatry. 166(6), 1995	Used mean maternal body weight during pregnancy, not body mass index

BMI, body mass index.

## Abbreviations

BMI: Body Mass Index
DSM: Diagnostic and Statistical Manual of Mental Disorders
ICD: International Classification of Disease
SD: Standard Deviation
WHO: World Health Organization
